# PDCoV NSP5 cleaves the selective autophagy receptor CCDC50 to disrupt autophagic degradation of the viral envelope protein

**DOI:** 10.1128/mbio.00259-26

**Published:** 2026-03-12

**Authors:** Ke Li, Hongyun Wei, Kangli Zhao, Dong Chen, Yu Sun, Peng Zhou, Hui Jin, Anan Jongkaewwattana, Sizhu Suolang, Dang Wang, Hongbo Zhou, Rui Luo

**Affiliations:** 1 State Key Laboratory of Agricultural Microbiology, College of Veterinary Medicine, Huazhong Agricultural University 47895, Wuhan, China; 2 Key Laboratory of Preventive Veterinary Medicine in Hubei Province, The Cooperative Innovation Center for Sustainable Pig Production, Wuhan, China; 3 Hubei Jiangxia Laboratory, Wuhan, China; 4 Virology and Cell Technology Research Team, National Center for Genetic Engineering and Biotechnology (BIOTEC), National Science and Technology Development Agency (NSTDA) 61191, Klong Nueng, Pathum Thani, Thailand; 5 College of Animal Science, Xizang Agricultural and Animal Husbandry University 667214 , Linzhi, China; The University of Iowa, Iowa City, Iowa, USA

**Keywords:** cleavage, CCDC50, NSP5, PDCoV, selective autophagy

## Abstract

Selective autophagy is a critical host defense mechanism that eliminates viral components through lysosomal degradation during coronavirus infection. Coronaviruses (CoVs), however, deploy countermeasures that disrupt this process, and several underlying mechanisms remain unresolved. Here, we identify the autophagy receptor CCDC50 as a substrate of the coronavirus-encoded NSP5 protease. During porcine deltacoronavirus (PDCoV) infection, NSP5 cleaves CCDC50 at glutamine 171 (Q171), a conserved site also processed by NSP5 from PEDV, TGEV, and SARS-CoV-2. Functionally, CCDC50 restricts PDCoV replication by recognizing the envelope (E) protein when it is modified with K63-linked polyubiquitin at lysine 72 (K72) and routing it for autophagic degradation, independently of canonical receptors such as SQSTM1/p62 and NBR1. NSP5-mediated cleavage disrupts CCDC50 interaction with LC3 and ubiquitin, reduces its capacity to target E for degradation, and thereby compromises its antiviral activity. Taken together, our study identifies CCDC50 as a previously uncharacterized antiviral autophagy receptor in coronavirus infection and reveals that PDCoV circumvents this defense through NSP5-mediated cleavage to promote productive infection.

**IMPORTANCE:**

In our study, we investigated the interplay between host autophagy pathways and coronavirus infection. We identified the selective autophagy receptor CCDC50 as a potent antiviral factor that suppresses porcine deltacoronavirus (PDCoV) replication. We demonstrated that CCDC50 specifically recognizes the viral envelope (E) protein and targets it for autophagic degradation, thereby restricting the virus. However, we also uncovered a sophisticated viral escape mechanism. We found that PDCoV’s main protease, NSP5, cleaves CCDC50 directly at a specific residue, glutamine 171. This proteolytic event impairs the ability of CCDC50 to interact with ubiquitin and the core autophagy machinery, effectively neutralizing its antiviral function and promoting viral replication. Significantly, we determined this to be a highly conserved strategy among coronaviruses. Our findings show that the NSP5 proteases of other divergent coronaviruses, including PEDV, TGEV, and even SARS-CoV-2, all target the same conserved site in CCDC50. These results reveal a common mechanism that coronaviruses use to subvert selective host autophagy.

## INTRODUCTION

Porcine deltacoronavirus (PDCoV) is an emerging enteropathogenic coronavirus (CoV) of the genus *Deltacoronavirus* in the family *Coronaviridae* (1). First identified in 2012 during molecular surveillance in Hong Kong, PDCoV was detected in diarrheic swine herds in the United States in 2014 and subsequently reported in Canada, South Korea, mainland China, and multiple Southeast Asian countries (2–4). Similar to other swine enteric CoVs, including porcine epidemic diarrhea virus (PEDV) and porcine transmissible gastroenteritis virus (TGEV), PDCoV causes severe diarrhea, vomiting, dehydration, and high mortality in neonatal piglets, resulting in major economic losses to the global swine industry (5). Beyond pigs, experimental or natural infections in calves, chickens, turkey poults, and mice indicate a broad host range (6–9). Of particular concern, detection of PDCoV in Haitian children with febrile illness highlights zoonotic potential and underscores the need to define host-virus interaction mechanisms that may influence cross-species transmission (10).

The ~25 kb positive-sense single-stranded RNA genome of PDCoV encodes the structural proteins spike (S), envelope (E), membrane (M), and nucleocapsid (N), as well as accessory proteins NS6 and NS7 and two overlapping replicase open reading frames (ORF1a and ORF1b) that give rise to polyproteins pp1a and pp1ab (3). These polyproteins are proteolytically processed by the papain-like protease (PLpro; NSP3) and the 3C-like protease (3CLpro; NSP5) to yield 15 nonstructural proteins (NSPs) essential for replication and transcription (11–13). In addition to viral polyprotein processing, NSP5 cleaves multiple host factors, including NEMO, STAT2, IFIT3, GSDMD, POLDIP3, and NBR1, thereby suppressing innate immune signaling, inhibiting pyroptosis, and modulating selective autophagy to favor replication (14–19).

Autophagy is an evolutionarily conserved degradative pathway that maintains cellular homeostasis by lysosomal recycling of damaged organelles, protein aggregates, and intracellular pathogens (20, 21). Beyond bulk turnover, selective autophagy targets defined cargos through receptor-mediated recognition (22). Canonical selective autophagy receptors such as SQSTM1/p62 (sequestosome 1), NBR1 (neighbor of BRCA1 gene 1), OPTN (optineurin), CALCOCO2/NDP52 (calcium-binding and coiled-coil domain-containing protein 2), TAX1BP1 (Tax1-binding protein 1), and TOLLIP (Toll-interacting protein) bridge ubiquitinated substrates to LC3-decorated autophagosomal membranes for lysosomal clearance (23–25). This pathway contributes to intrinsic antiviral defense by degrading viral components critical for replication or immune evasion. For example, SQSTM1/p62 mediates the autophagic degradation of the Seneca Valley virus (SVV) VP1 and VP3 protein to limit viral replication (26); NBR1 targets the PDCoV E protein for degradation (19); and CALCOCO2/NDP52 restricts PEDV by promoting the clearance of its N protein (27). These findings establish selective autophagy as a key host strategy for restricting diverse viral infections.

CCDC50 (coiled-coil domain containing 50) has recently been characterized as a selective autophagy receptor implicated in the regulation of innate immune signaling (28, 29). It comprises an N-terminal coiled-coil (CC) domain, two C-terminal ubiquitin-interacting MIU motifs, and an LC3-interacting region (LIR) embedded within MIU1 (28). In contrast to classical selective autophagy receptors such as SQSTM1/p62 and NBR1, which engage LC3 predominantly through the canonical LIR motifs and recognize ubiquitinated cargos via dedicated ubiquitin-associated (UBA) domains, CCDC50 displays a distinct mode of interaction with the autophagy machinery. Notably, CCDC50 engages LC3 not only through the canonical LC3 docking site (LDS) but also via an alternative ubiquitin-interacting motif docking site (UDS), thereby forming a dual-interface interaction with LC3 proteins (28). Although CCDC50 lacks canonical UBA domains comparable to those of SQSTM1 or NBR1, it has been shown to recognize K63-linked polyubiquitinated substrates through its MIU motifs and to recruit these cargos to autophagosomes for lysosomal degradation. Recent studies show that CCDC50 directs innate immune signaling proteins such as RIG-I, MDA5, and STING to autophagic degradation, thereby modulating type I interferon responses (28, 29). However, whether CCDC50 directly recognizes and degrades viral structural components has not been established.

In this study, we show that CCDC50 suppresses PDCoV replication by directly interacting with the viral E protein and promoting its autophagic degradation independently of SQSTM1 and NBR1. We further demonstrate that the PDCoV main protease NSP5 specifically cleaves CCDC50 at Q171, impairing its ability to mediate selective degradation of E. Notably, NSP5 homologs from PEDV, TGEV, and SARS-CoV-2 also target the same cleavage site in CCDC50. These findings uncover an antiviral function of CCDC50 in selective autophagic clearance of a coronavirus structural protein and reveal a convergent protease-mediated strategy by which coronaviruses antagonize host receptor-guided autophagy to enhance infection.

## RESULTS

### PDCoV infection induces proteolytic cleavage of CCDC50

**Fig 1:**
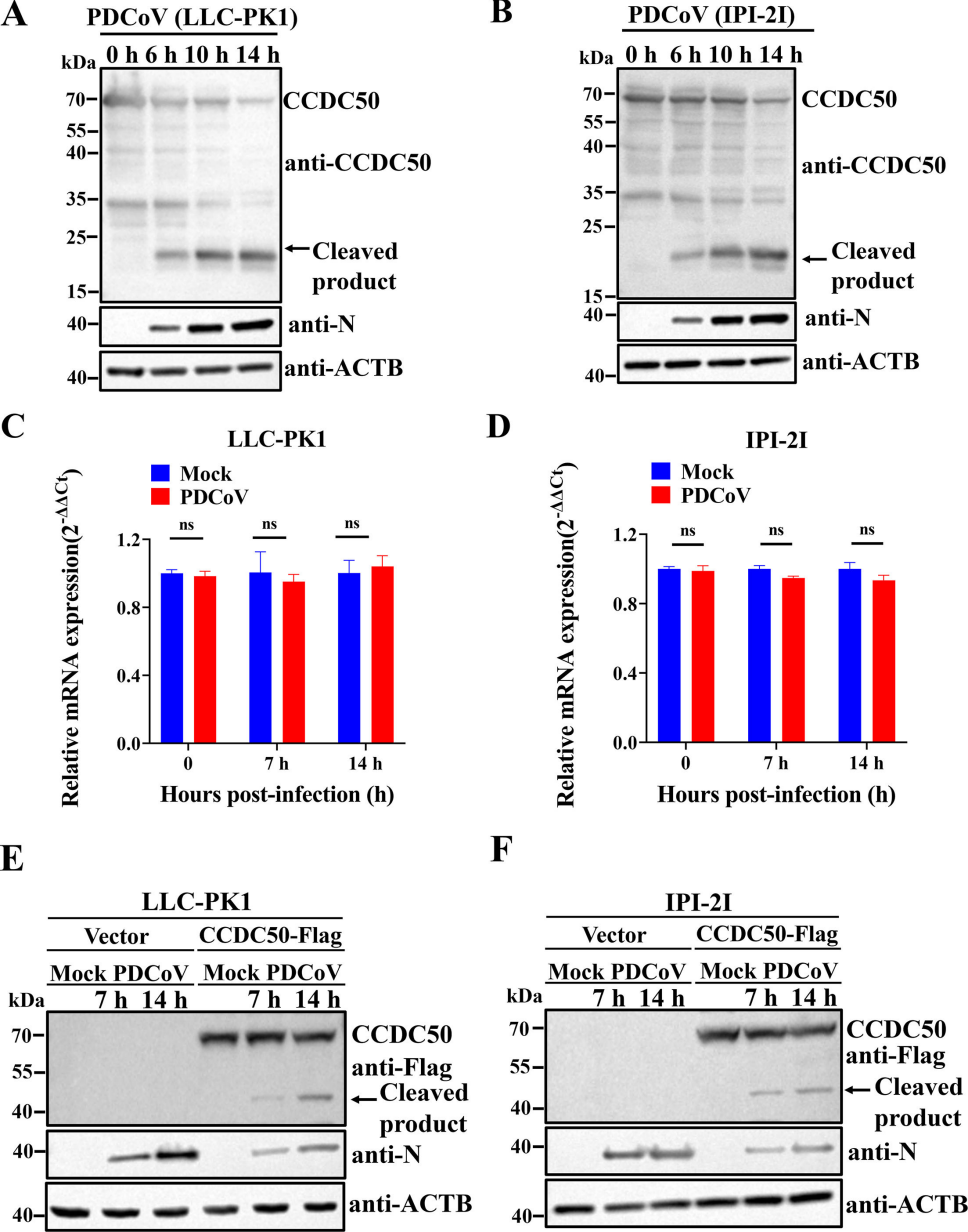
PDCoV infection induces cleavage of CCDC50. (**A, B**) LLC-PK1 (**A**) and IPI-2I (**B**) cells were infected with PDCoV, and endogenous CCDC50 protein levels were assessed by Western blotting with an anti-CCDC50 antibody at 6, 10, and 14 h p.i. (**C, D**) CCDC50 mRNA levels in PDCoV-infected LLC-PK1 (**C**) and IPI-2I (**D**) cells were quantified by RT-qPCR. (**E, F**) LLC-PK1 (**E**) and IPI-2I (**F**) cells were transfected with Flag-tagged CCDC50 and then infected with PDCoV for 7 or 14 h. Exogenous CCDC50 expression was detected by Western blotting with an anti-Flag antibody targeting the C-terminus. Data represent the mean ± SD of three replicates. ns, not significant.

We first assessed whether PDCoV infection alters CCDC50 protein abundance. In LLC-PK1 and IPI-2I cells infected with PDCoV, Western blot using an antibody recognizing the N terminus of CCDC50 showed a time-dependent loss of the full-length (FL) protein accompanied by the emergence of a prominent ~20 kDa N-terminal fragment (Fig. 1A and B). This pattern is consistent with virus-induced proteolytic cleavage of CCDC50 rather than simple downregulation. To determine whether the reduction of FL CCDC50 reflected decreased transcription, CCDC50 mRNA levels were quantified by RT-qPCR. PDCoV infection did not significantly change CCDC50 transcript abundance in either cell line (Fig. 1C and D), indicating a post-transcriptional mechanism. To confirm that the observed fragments derive from CCDC50 and to visualize both termini, we ectopically expressed CCDC50 bearing a C-terminal Flag tag in LLC-PK1 and IPI-2I cells and subsequently infected the cells with PDCoV. Western blot with an anti-Flag antibody detected a ~45 kDa C-terminal fragment along with reduced FL protein levels (Fig. 1E and F), corroborating PDCoV-induced proteolysis of CCDC50. Collectively, these data demonstrate that PDCoV infection triggers CCDC50 cleavage, generating an approximately 20 kDa N-terminal fragment without reducing CCDC50 mRNA expression.

### PDCoV NSP5 cleaves CCDC50 at residue Q171

**Fig 2:**
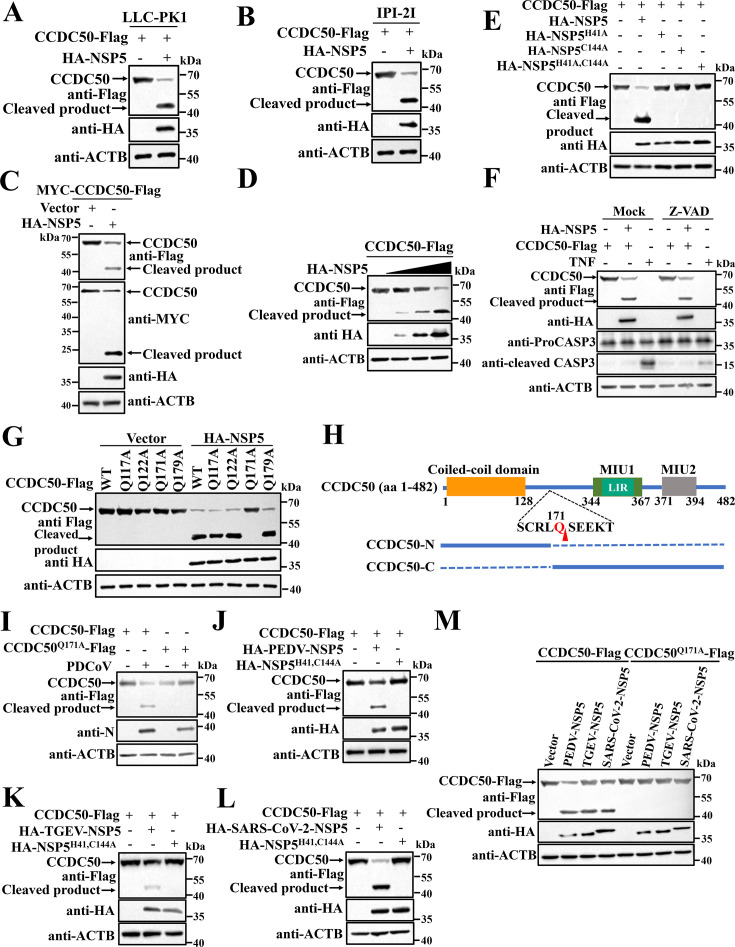
PDCoV NSP5 cleaves CCDC50 at Q171 via its protease activity. (**A, B**) LLC-PK1 (**A**) and IPI-2I (**B**) cells were co-transfected with CCDC50-Flag and HA-NSP5 plasmids. At 28 h post-transfection, CCDC50 expression was analyzed by Western blotting. (**C**) HEK-293T cells were co-transfected with MYC-CCDC50-Flag and HA-NSP5. The N- and C-terminal fragments were detected with anti-MYC and anti-Flag antibodies, respectively. (**D**) Dose-dependent cleavage of CCDC50 by HA-NSP5 was examined in HEK-293T cells by immunoblotting. (**E**) HEK-293T cells were co-transfected with CCDC50-Flag and either WT or catalytically inactive NSP5 mutants (H41A, C144A, or H41A/C144A). CCDC50 cleavage was analyzed by Western blotting. (**F**) HEK-293T cells co-expressing CCDC50-Flag and HA-NSP5 were treated with Z-VAD-FMK (20 μM) or mock for 12 h before lysis. Cell lysates were analyzed by Western blotting with the indicated antibodies. TNF-α (20 ng/mL) was used to confirm the inhibition of caspase-3 cleavage by Z-VAD-FMK. (**G**) CCDC50-Flag or cleavage site mutants were transfected into HEK-293T cells together with HA-NSP5. Cleavage efficiency was determined by Western blotting. (**H**) Schematic representation of CCDC50 domains and NSP5-mediated cleavage products. (**I**) LLC-PK1 cells transfected with WT CCDC50 or the Q171A mutant were infected with PDCoV for 14 h. CCDC50 expression was analyzed by Western blotting. (**J–L**) CCDC50-Flag was co-expressed with NSP5 or catalytically inactive NSP5 mutants of PEDV (**J**), TGEV (**K**), or SARS-CoV-2 (**L**) in HEK-293T cells. Cleavage was assessed by Western blotting. (**M**) WT or Q171A mutant CCDC50 was co-transfected with NSP5s from PEDV, TGEV, or SARS-CoV-2 in HEK-293T cells. The cleavage of CCDC50 was analyzed by Western blotting.

Coronavirus NSP5 (3C-like protease) processes viral polyproteins and selected host substrates to promote viral replication (30, 31). To test whether PDCoV NSP5 mediates CCDC50 cleavage, Flag-tagged CCDC50 was co-expressed with HA-tagged NSP5 in LLC-PK1 and IPI-2I cells. Western blot analysis revealed a reproducible ~45 kDa C-terminal fragment together with reduction of full-length CCDC50 (Fig. 2A and B), indicating NSP5-dependent processing. To map fragment termini, a dual-tagged construct (MYC-CCDC50-Flag) was co-expressed with NSP5 in HEK-293T cells. Two discrete products were detected: a ~20 kDa N-terminal fragment (anti-MYC) corresponding to the species observed in infected cells and a ~45 kDa C-terminal fragment (anti-Flag) (Fig. 2C). Increasing NSP5 expression produced a dose-dependent increase in cleavage (Fig. 2D). Protease dependence was confirmed by co-expression with catalytically inactive NSP5 mutants (H41A, C144A, or double H41A/C144A), none of which generated cleavage fragments (Fig. 2E). Inhibition of caspases with Z-VAD-FMK did not affect fragment formation (Fig. 2F), excluding caspase-mediated processing.

To identify the NSP5 cleavage site, we considered coronavirus NSP5 specificity, which requires glutamine (Q) at the P1 position (19, 31), and the observed fragment sizes. Four candidate glutamine residues (Q117, Q122, Q171, and Q179) were individually mutated to alanine. Only the Q171A mutation abolished cleavage, whereas Q117A, Q122A, and Q179A remained susceptible (Fig. 2G and H). Consistently, PDCoV infection failed to induce cleavage of CCDC50^Q171A^, while wild-type CCDC50 was efficiently processed (Fig. 2I), identifying Q171 as the main PDCoV NSP5 cleavage site.

To assess conservation across coronaviruses, CCDC50 was co-expressed with wild-type or catalytically inactive NSP5 enzymes from PEDV, TGEV, and SARS-CoV-2. Each wild-type NSP5 induced CCDC50 cleavage, whereas the inactive counterparts did not (Fig. 2J through L). Interestingly, CCDC50^Q171A^ resisted cleavage by all NSP5 homologs tested (Fig. 2M), indicating that Q171 constitutes a conserved NSP5 target site across these divergent coronaviruses. Collectively, these findings demonstrate that coronavirus NSP5 proteases cleave CCDC50 at residue Q171 via a conserved proteolytic mechanism.

### CCDC50 inhibits PDCoV propagation

**Fig 3:**
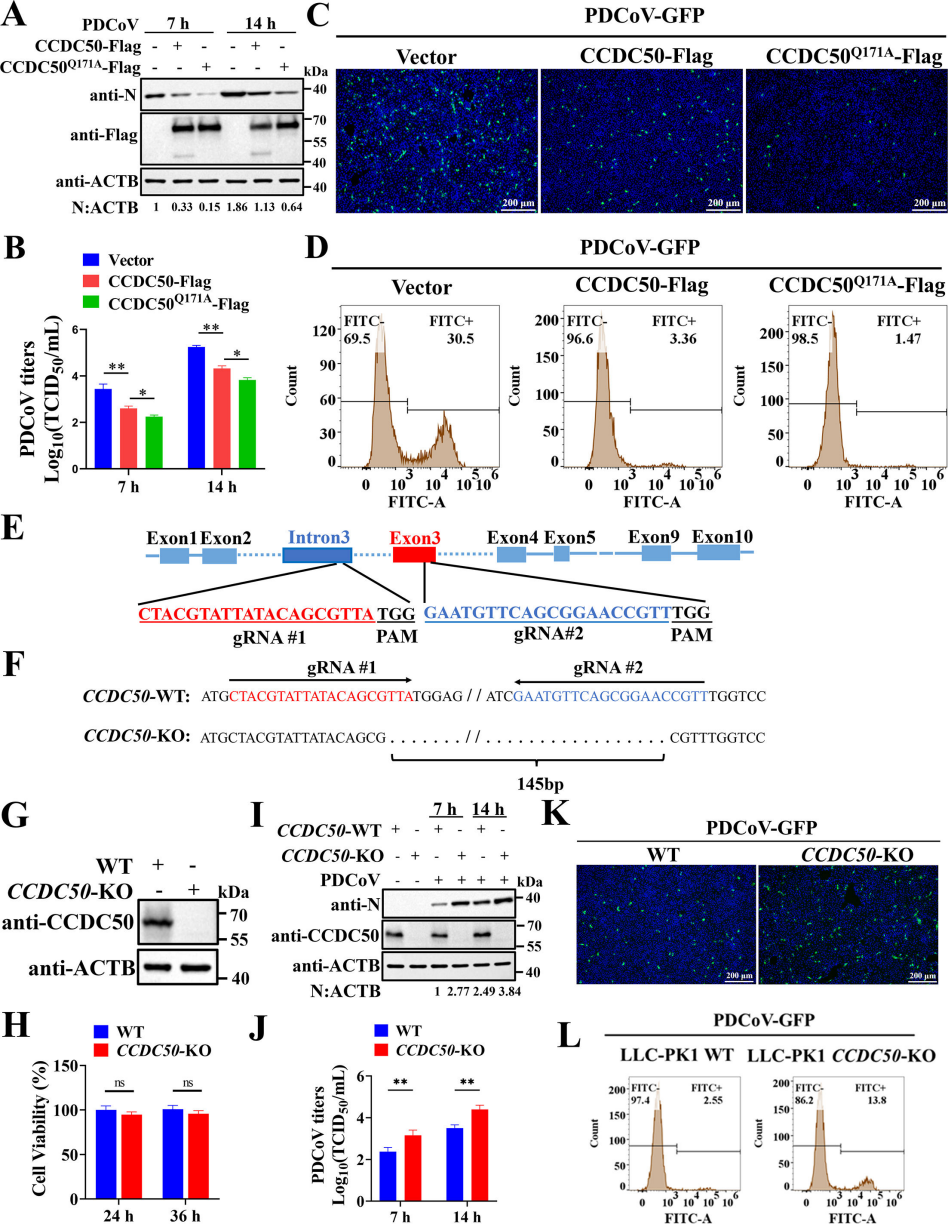
CCDC50 inhibits PDCoV propagation. (**A, B**) LLC-PK1 cells were transfected with plasmids expressing CCDC50-Flag, CCDC50^Q171A^-Flag, or an empty vector, followed by PDCoV infection. At 7 and 14 h p.i., cells were harvested for Western blotting (**A**), and culture supernatants were collected for TCID_50_ assays (**B**). (**C, D**) LLC-PK1 cells were transfected with plasmids expressing CCDC50-Flag, CCDC50Q171A-Flag, or an empty vector, then infected with PDCoV-GFP for 14 h. GFP fluorescence was analyzed by confocal microscopy (**C**) and flow cytometry (**D**). Scale bar, 200 μm. (**E**) Schematic diagram of the *CCDC50* knockout (KO) strategy. (**F, G**) Validation of *CCDC50*-KO in LLC-PK1 cells by DNA sequencing (**F**) and Western blotting (**G**). (**H**) Cell viability of WT and *CCDC50*-KO LLC-PK1 cells. (**I, J**) WT and *CCDC50*-KO cells were infected with PDCoV, and samples were collected at the indicated time points for Western blotting (**I**) and TCID_50_ assays (**J**). (**K, L**) WT and *CCDC50*-KO cells were infected with PDCoV-GFP for 7 h and analyzed by confocal microscopy (**K**) and flow cytometry (**L**). Scale bar, 200 μm. Results from TCID_50_ (**B, J**) and cell viability (**H**) assays are presented as mean ± SD of three independent experiments. Statistical significance was determined using Student’s *t*-test. * *P* < 0.05; ** *P* < 0.01; ns, not significant.

We next evaluated the functional impact of CCDC50 on PDCoV replication. Ectopic expression of wild-type CCDC50 in LLC-PK1 cells markedly reduced viral N protein levels and infectious titers at 7 and 14 h post-infection (h p.i.), and the cleavage-resistant mutant CCDC50^Q171A^ exerted an even stronger inhibitory effect (Fig. 3A and B). Consistently, using a recombinant GFP-expressing PDCoV (PDCoV-GFP), both fluorescence microscopy and flow cytometry demonstrated a pronounced decrease in the proportion and mean fluorescence intensity of infected cells upon expression of CCDC50, with further reduction by CCDC50^Q171A^ (Fig. 3C and D). To corroborate these findings via loss-of-function analysis, *CCDC50*-knockout (*CCDC50*-KO) LLC-PK1 cells were generated using CRISPR-Cas9. Sequencing confirmed a 145-nucleotide deletion spanning intron 3 and exon 3 (Fig. 3E and F), and Western blot verified complete absence of CCDC50 protein (Fig. 3G). CCK-8 assays showed comparable viability between wild-type (WT) and *CCDC50*-KO cells (Fig. 3H), excluding cytotoxic effects of the knockout. Relative to WT cells, *CCDC50*-KO cells exhibited elevated PDCoV N protein accumulation (Fig. 3I), increased viral titers (Fig. 3J), and enhanced PDCoV-GFP replication by microscopy and flow cytometry (Fig. 3K and L). Taken together, these gain- and loss-of-function data demonstrate that CCDC50 restricts PDCoV replication, and that preventing NSP5-mediated cleavage (Q171A) potentiates its antiviral activity.

### CCDC50 targets PDCoV E for degradation

**Fig 4:**
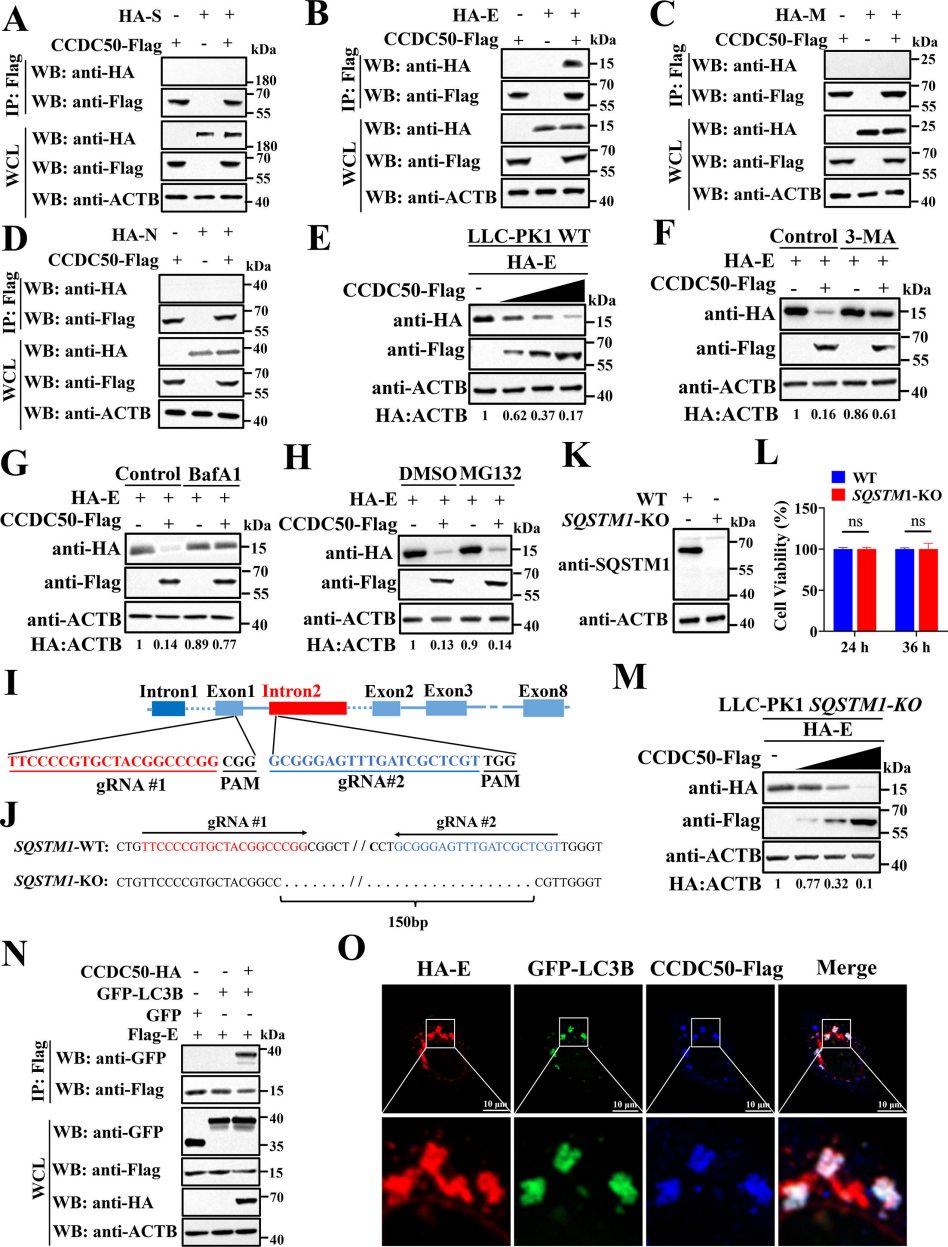
CCDC50 interacts with PDCoV E and mediates its autophagic degradation. (**A–D**) HEK-293T cells were co-transfected with CCDC50-Flag and HA-tagged S, E, M, or N. At 28 h post-transfection, cell lysates were immunoprecipitated with anti-Flag antibodies and analyzed by Western blotting. (**E**) LLC-PK1 cells were transfected with increasing amounts of CCDC50-Flag together with HA-E, and protein levels were examined by Western blotting. (**F–H**) LLC-PK1 cells were co-transfected with HA-E and either CCDC50-Flag or empty vector. At 18 h post-transfection, cells were treated with DMSO or 3-MA (10 mM) (**F**), Baf A1 (10 µM) (**G**), or MG132 (10 μM) (**H**) for 6 h and analyzed by Western blotting. (**I**) Schematic representation of the *SQSTM1*-KO strategy. (**J, K**) Validation of *SQSTM1*-KO in LLC-PK1 cells by Sanger sequencing (**J**) and Western blotting (**K**). (**L**) Cell viability of WT and *SQSTM1*-KO LLC-PK1 cells. Data are expressed as mean ± SD from three independent experiments. Statistical significance was determined using Student’s *t*-test. ns, not significant. (**M**) *SQSTM1*-KO cells were transfected with increasing amounts of CCDC50-Flag together with HA-E. Protein levels were determined by Western blotting. (**N**) HEK-293T cells were co-transfected with CCDC50-HA, GFP-LC3B, and Flag-E for 28 h. Cell lysates were subjected to immunoprecipitation with an anti-Flag antibody and then subjected to Western blotting. (**O**) HeLa cells were co-transfected with CCDC50-Flag, GFP-LC3B, and HA-E for 24 h. Cells were fixed and subjected to immunofluorescence staining with anti-Flag and anti-HA antibodies. Scale bar: 10 μm. ns, not significant.

CCDC50 is a selective autophagy receptor that mediates lysosomal degradation of polyubiquitinated cargo (32). We therefore asked whether it targets porcine PDCoV structural proteins for autophagic clearance. Co-immunoprecipitation (co-IP) assays showed that CCDC50 specifically associated with the E protein, but not with S, M, or N proteins (Fig. 4A through D). To test whether CCDC50 promotes E degradation, LLC-PK1 cells were co-transfected with E and increasing amounts of CCDC50. Immunoblotting revealed a dose-dependent reduction in E abundance (Fig. 4E). Pharmacologic pathway discrimination demonstrated that inhibition of autophagy initiation with 3-methyladenine (3-MA) or blockade of autophagic flux with bafilomycin A1 (Baf A1) restored E levels, whereas proteasome inhibition with MG132 did not (Fig. 4F through H), indicating that CCDC50 mediates E degradation via the autophagy-lysosome pathway rather than the ubiquitin-proteasome system.

Because SQSTM1/p62 is a canonical autophagy receptor involved in the degradation of PDCoV components (33), we examined whether CCDC50 acts independently of them. CRISPR-Cas9-mediated deletion generated *SQSTM1*-knockout (*SQSTM1*-KO) LLC-PK1 cells, confirmed by a 150 nt deletion and complete loss of SQSTM1 protein (Fig. 4I through K). Cell viability was unaffected (Fig. 4L). CCDC50 still reduced E protein levels in *SQSTM1*-KO cells (Fig. 4M), indicating that CCDC50-mediated E degradation does not require SQSTM1.

Our previous work demonstrated that the selective autophagy receptor NBR1 mediates autophagic degradation of the PDCoV E protein (19). To determine whether the antiviral activity of CCDC50 is independent of NBR1 or requires NBR1, we generated *NBR1/CCDC50* double-knockout LLC-PK1 cells using CRISPR-Cas9. Sequencing analysis revealed a single-nucleotide deletion in CCDC50 and a single-nucleotide insertion in NBR1, and Western blotting confirmed complete loss of both proteins (Fig. S1A through E). Cell viability was comparable between WT and *CCDC50/NBR1*-KO cells (Fig. S1F). Re-expression of either NBR1 or CCDC50 in *NBR1*/*CCDC50* double-knockout cells resulted in dose-dependent degradation of the PDCoV E protein (Fig. S1G and H). Consistently, Western blotting and TCID_50_ assays showed that deletion of either NBR1 or CCDC50 individually enhanced PDCoV replication, whereas simultaneous loss of both receptors led to a substantially greater increase in viral replication than either single knockout (Fig. S1I and J). Moreover, immunofluorescence and flow cytometry analyses using PDCoV-GFP further confirmed higher levels of viral replication in *CCDC50*/*NBR1* double-knockout cells compared with either *CCDC50*-KO or *NBR1*-KO cells (Fig. S1K and L). Taken together, these data demonstrate that CCDC50 restricts PDCoV infection by promoting E protein degradation independently of both SQSTM1 and NBR1.

To assess whether CCDC50 facilitates recruitment of E protein to autophagosomes, we evaluated E-LC3B association in the presence or absence of ectopic CCDC50. In cells lacking exogenous CCDC50, E-LC3B interaction was undetectable; CCDC50 overexpression markedly enhanced their co-IP (Fig. 4N). Confocal microscopy further revealed robust co-localization of E with CCDC50 and LC3B puncta (Fig. 4O). Taken together, these results identify CCDC50 as a molecular adaptor that links the PDCoV E protein to LC3B, driving its selective autophagic degradation independently of SQSTM1 and NBR1.

### CCDC50 recognizes K63-linked ubiquitination of the PDCoV E protein

**Fig 5:**
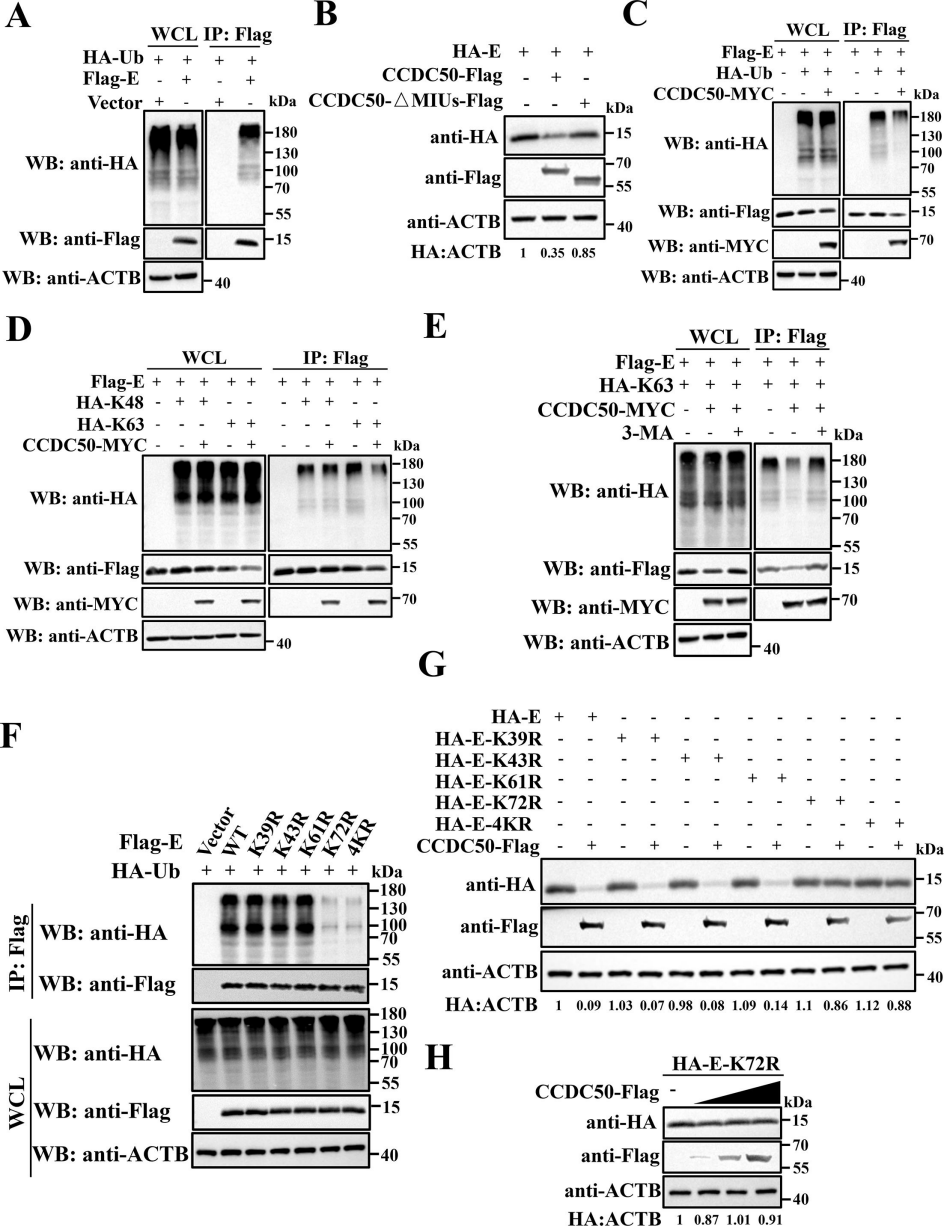
CCDC50 recognizes the K63-linked ubiquitination of the PDCoV E protein. (**A**) HEK-293T cells were co-transfected with Flag-E and HA-ubiquitin (HA-Ub) plasmids. At 28 h post-transfection, cell lysates were immunoprecipitated with anti-Flag antibody and analyzed by Western blotting. (**B**) Western blot analysis of E protein levels in LLC-PK1 cells expressing WT CCDC50 or a MIU-deletion mutant. (**C**) HEK-293T cells were co-transfected with Flag-E, HA-Ub, and either CCDC50-MYC or an empty vector. At 28 h post-transfection, ubiquitination of E protein was analyzed by immunoprecipitation followed by immunoblotting. (**D**) HEK-293T cells were co-transfected with Flag-E and either HA-K48-Ub or HA-K63-Ub. Polyubiquitination of E was analyzed by co-IP and Western blotting. (**E**) HEK-293T cells were co-transfected with Flag-E and HA-K63-Ub, and treated with or without 3-MA (10 mM) for 6 h. Lysates were analyzed by co-IP and Western blotting. (**F**) HEK-293T cells were co-transfected with HA-Ub and either WT or lysine-to-arginine (K-to-R) mutants of Flag-E. Ubiquitination of E was analyzed by co-IP and immunoblotting. (**G**) LLC-PK1 cells were co-transfected with HA-tagged WT or K-to-R mutants of E protein and CCDC50-Flag. Protein levels were analyzed by Western blotting. (**H**) LLC-PK1 cells were co-transfected with increasing amounts of CCDC50-Flag and HA-E-K72R. Protein levels were analyzed by Western blotting.

Because CCDC50 contains tandem MIU domains, we asked whether its promotion of PDCoV E protein degradation depends on E ubiquitination. Co-IP confirmed that E protein is ubiquitinated (Fig. 5A). An MIU-deleted mutant (CCDC50-ΔMIUs) failed to reduce E abundance (Fig. 5B), suggesting that ubiquitin binding is required for CCDC50-mediated E degradation. Overexpression of CCDC50 decreased both total E protein and its ubiquitinated species (Fig. 5C). Consistent with the reported preference of CCDC50 for K63-linked chains (28, 29, 34), CCDC50 selectively reduced K63-, but not K48-, polyubiquitinated E (Fig. 5D), and inhibition of autophagy initiation with 3-methyladenine (3-MA) restored K63-linked ubiquitinated E levels (Fig. 5E), implicating the autophagy-lysosome pathway.

To define the ubiquitination site(s) required for this process, four single lysine-to-arginine (K to R) mutants and a quadruple lysine mutant (E-4KR) were generated. Co-IP analysis showed markedly diminished ubiquitination of K72R and E-4KR (Fig. 5F). Accordingly, CCDC50 failed to promote degradation of either E-K72R or E-4KR (Fig. 5G), and increasing expression of CCDC50 did not reduce K72R abundance (Fig. 5H). These data altogether identify K72 as a critical ubiquitination site whose K63-linked modification enables CCDC50-dependent selective autophagic degradation of the PDCoV E protein.

### NSP5-mediated cleavage of CCDC50 compromises its selective autophagy function

**Fig 6:**
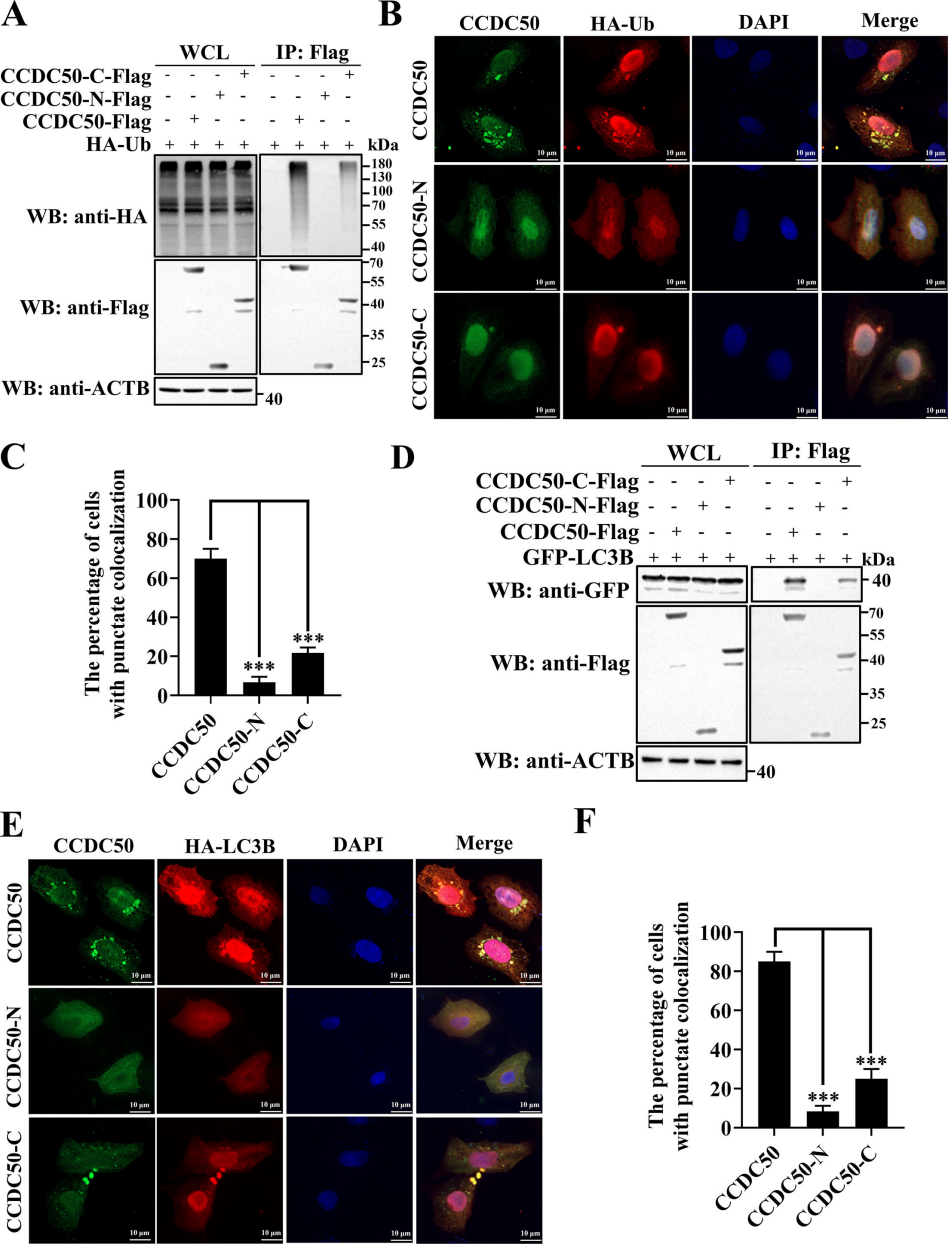
Cleavage of CCDC50 impairs its function in selective autophagy. (**A, D**) HEK-293T cells were co-transfected with plasmids expressing empty vector, CCDC50-Flag, CCDC50-N-Flag, or CCDC50-C-Flag, together with HA-Ub (**A**) or GFP-LC3B (**D**). At 28 h post-transfection, cell lysates were subjected to immunoprecipitation with anti-Flag antibodies, followed by Western blotting with the indicated antibodies. (**B, E**) HeLa cells were co-transfected with plasmids encoding CCDC50-Flag, CCDC50-N-Flag, or CCDC50-C-Flag, together with HA-Ub (**B**) or HA-LC3B (**E**). At 28 h post-transfection, cells were fixed and stained with anti-Flag and anti-HA antibodies for immunofluorescence analysis. The fluorescence signals were visualized by confocal microscopy. Scale bar, 10 μm. (**C, F**) Quantification of cells showing punctate colocalization between CCDC50 or its cleavage products and either HA-Ub (**C**) or HA-LC3B (**F**). At least 20 cells were analyzed per experiment from three independent replicates. Data are expressed as mean ± SD. Statistical significance was determined using Student’s *t*-test: *** *P* < 0.001.

We next investigated whether proteolytic cleavage impairs the adaptor activity of CCDC50 by comparing the ubiquitin and LC3B binding capacities of full-length CCDC50 and its NSP5-derived fragments. Co-IP showed robust association of full-length CCDC50 with ubiquitin and LC3B, reduced binding by the C-terminal fragment (CCDC50-C), and no detectable interaction by the N-terminal fragment (CCDC50-N), which lacks both MIU and LIR motifs (Fig. 6A and D). Confocal microscopy confirmed these results: wild-type CCDC50 exhibited extensive punctate co-localization with ubiquitin (≈70%) and LC3B (≈85%), whereas CCDC50-C displayed diminished overlap and CCDC50-N showed minimal co-localization (Fig. 6B, C, E and F). These results indicate that NSP5-mediated cleavage disrupts the modular architecture required for simultaneous ubiquitin and LC3B engagement, thereby compromising CCDC50’s role in selective autophagic cargo delivery.

### NSP5-mediated cleavage fragments attenuate CCDC50 antiviral activity

**Fig 7:**
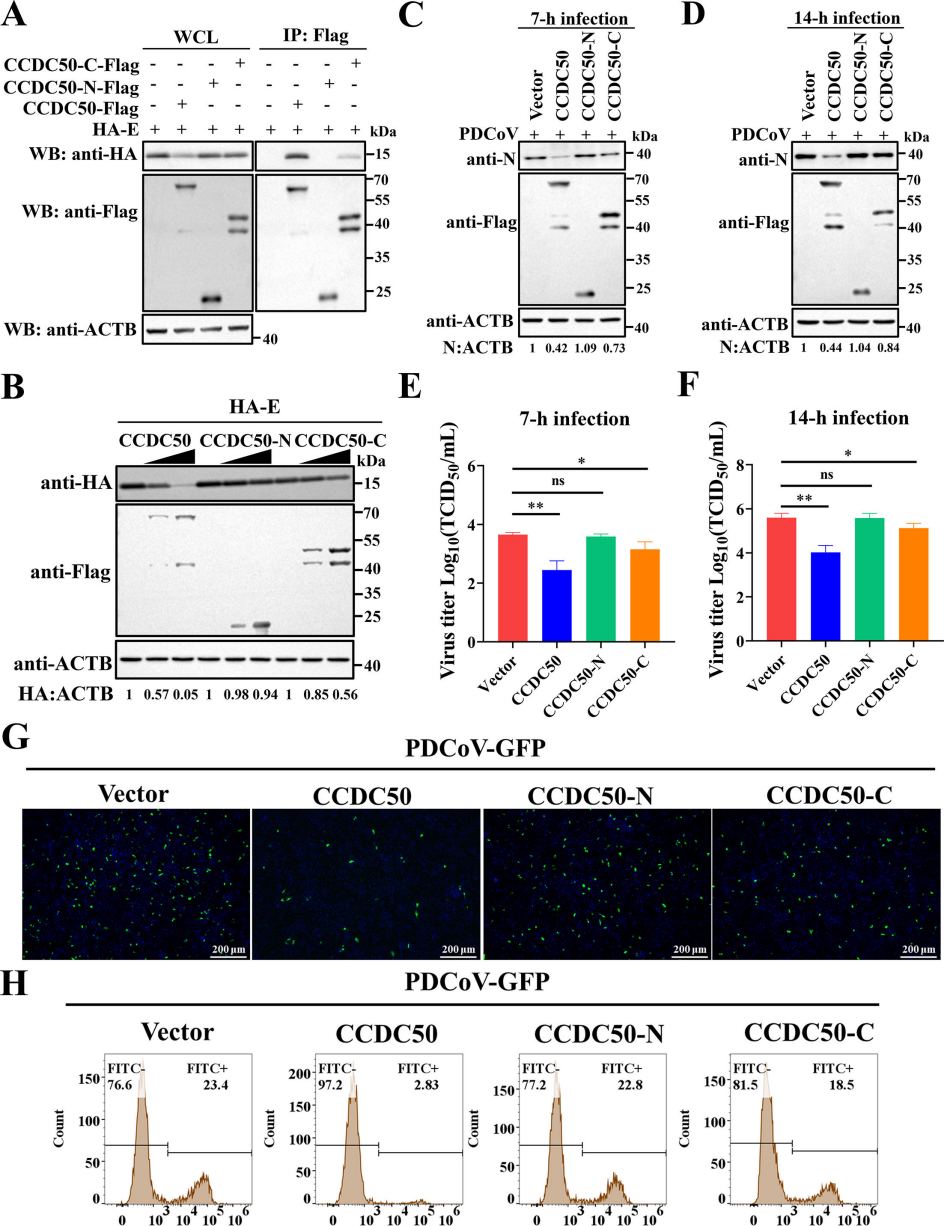
Cleavage of CCDC50 attenuates its antiviral activity. (**A**) HEK-293T cells were co-transfected with plasmids encoding HA-E and either empty vector, CCDC50-Flag, CCDC50-N-Flag, or CCDC50-C-Flag. At 28 h post-transfection, cell lysates were subjected to immunoprecipitation with anti-Flag antibodies and analyzed by Western blotting. (**B**) LLC-PK1 cells were co-transfected with HA-E and increasing amounts of CCDC50-Flag, CCDC50-N-Flag, or CCDC50-C-Flag. Protein expression was determined by Western blotting. (**C–F**) LLC-PK1 cells were transfected with the indicated plasmids and infected with PDCoV. At 7 and 14 h p.i., cell lysates and supernatants were collected for Western blotting (**C, D**) and TCID_50_ assays (**E, F**). (**G, H**) LLC-PK1 cells were transfected with the indicated plasmids and infected with PDCoV-GFP for 12 h. GFP signals were analyzed by fluorescence microscopy (**G**) and flow cytometry (**H**). Scale bar, 200 μm. Results of TCID50 assays are expressed as mean ± SD of three independent experiments. Statistical significance was determined using Student’s *t*-test: * *P* < 0.05; ** *P* < 0.01; ns, not significant.

We next asked whether NSP5-generated CCDC50 fragments retain the capacity to interact with the PDCoV E protein. Co-IP analysis showed that CCDC50-N failed to bind E, whereas CCDC50-C retained a weak yet markedly reduced interaction compared with full-length CCDC50 (Fig. 7A). Correspondingly, in LLC-PK1 cells co-transfected with HA-tagged E and increasing amounts of each construct, wild-type (WT) CCDC50 induced a clear dose-dependent decrease in E protein abundance; CCDC50-N completely lost this activity, while CCDC50-C mediated only a modest reduction (Fig. 7B). To determine functional consequences for viral replication, cells expressing WT or truncated CCDC50 were infected with PDCoV and analyzed at 7 and 14 h p.i. Relative to WT CCDC50, CCDC50-N failed to diminish viral N protein levels or infectious titers, indicating complete loss of antiviral function, whereas CCDC50-C conferred only partial suppression (Fig. 7C through F). Consistently, fluorescence microscopy and flow cytometry using PDCoV-GFP demonstrated no detectable impact of CCDC50-N and only limited inhibition by CCDC50-C (Fig. 7G and H). Taken together, these results show that NSP5-mediated cleavage attenuates the antiviral activity of CCDC50 by separating domains required for high-affinity E recognition and efficient degradation, yielding fragments with absent (N-terminal) or diminished (C-terminal) restrictive capacity.

## DISCUSSION

Coronavirus replication is tightly regulated by the host autophagy machinery, which performs a dual and seemingly contradictory role by both facilitating viral replication and mediating antiviral defense (35, 36). While autophagy facilitates viral replication by providing double-membrane vesicles that serve as replication organelles, it also functions as a host defense mechanism by targeting viral components for lysosomal degradation. Selective autophagy receptors, such as SQSTM1/p62, NBR1, OPTN, and TOLLIP, are increasingly recognized for their antiviral functions by mediating the clearance of ubiquitinated viral proteins by autophagy. For example, SQSTM1/p62 targets the SARS-CoV-2 M protein for autophagic degradation, while TOLLIP promotes the removal of ACE2, the host receptor for viral entry, thereby limiting SARS-CoV-2 infection (37, 38). Here, we identified CCDC50 as a previously uncharacterized selective autophagy receptor that suppresses PDCoV replication by recognizing K63-linked polyubiquitin chains on the E protein and targeting it for autophagic degradation. Specifically, the protease NSP5 encoded by PDCoV cleaves CCDC50 at a conserved glutamine residue (Q171), impairing its interaction with LC3 and ubiquitin, weakening its ability to recognize the viral E protein and significantly reducing its antiviral activity. These results reveal a novel mechanism to bypass the coronavirus immune system and unveil a previously unrecognized antiviral function of CCDC50 as a selective autophagy receptor.

CCDC50 is a recently identified autophagic cargo receptor that regulates innate immune responses by targeting key signaling molecules for degradation. It selectively binds K63-linked polyubiquitinated forms of RIG-I, MDA5, and STING, promoting their autophagic clearance and attenuating type I interferon signaling during RNA or DNA virus infection (28, 29). However, its direct involvement in antiviral defense has not been previously reported. Here, we show that CCDC50 recognizes the K63-linked polyubiquitinated PDCoV-E protein and facilitates its selective autophagic degradation, thereby restricting viral replication. We identified K72 as the major ubiquitination site on the E protein required for CCDC50-mediated degradation. Remarkably, K72 is highly conserved among PDCoV strains (Fig. S2), suggesting that autophagic targeting of this site represents a conserved host strategy for restricting viral replication. This substrate preference is consistent with the demonstrated selectivity of CCDC50 for K63-linked ubiquitin chains (28, 39). Comparable examples include SQSTM1/p62, which targets the K63-ubiquitinated VP2 capsid protein of infectious bursal disease virus (IBDV) for degradation (40), while TOLLIP and NDP52 recognize the K48-linked ubiquitinated dengue virus (DENV) NS3 and PDCoV N proteins, respectively, for autophagic degradation (41, 42). Our previous work has shown that NBR1 restricts PDCoV replication by targeting the E protein via a ubiquitin-independent mechanism (19). Furthermore, CCDC50-mediated degradation of E protein remains unaffected in cells deficient in NBR1 or SQSTM1/p62, suggesting that CCDC50 degrades E protein independently of these receptors. Taken together, these findings support a broader model in which distinct selective autophagy receptors act through complementary, non-overlapping mechanisms to reinforce host antiviral defense.

Coronavirus NSP5 is a cysteine protease that promotes viral replication not only by processing viral polyproteins but also by cleaving antiviral host factors (43). For example, NSP5 interferes with the innate immune response in SARS-CoV-2 by targeting key components such as RIG-I, NEMO (NF-κB essential modulator), and TAB1 (TGF-beta-activated kinase 1 binding protein 1) (44–46). NSP5 not only impairs the interferon response but also undermines the selective autophagy-mediated antiviral defense by cleaving autophagy receptors that mediate the degradation of viral components. For example, in SARS-CoV-2, NSP5 cleaves SQSTM1/p62, thereby impairing autophagic clearance of the viral M protein (37), while our previous work showed that in PDCoV, NSP5 targets NBR1 to bypass autophagic degradation of the E protein (19). Here, we identify CCDC50 as another target of coronavirus NSP5. We show that NSP5 of PDCoV cleaves CCDC50 at a conserved Q171 site, and that NSP5 of PEDV, TGEV, and SARS-CoV-2 also cleave CCDC50 at this site, suggesting a conserved viral strategy to disable CCDC50-mediated antiviral autophagy. This cleavage generates two fragments, CCDC50-N, which contains the N-terminal coiled-coil domain, and CCDC50-C, which comprises the MIU1 and MIU2 motifs required for LC3 and K63-linked ubiquitin binding, respectively. In agreement with previous studies, we found that CCDC50-C retains LC3 and ubiquitin binding, whereas CCDC50-N completely loses these interactions (28). However, compared to full-length CCDC50, the binding capacity of CCDC50-C is markedly reduced, likely due to impaired oligomerization mediated by the N-terminal coiled-coil domain. Fluorescence microscopy also revealed reduced puncta formation by the cleavage fragments, supporting the notion that oligomerization of CCDC50 facilitates stable binding with LC3 and ubiquitinated cargo (28). Since CCDC50 recognizes the K63-linked ubiquitinated PDCoV E protein, cleavage by NSP5 also disrupts its ability to bind E, thereby attenuating its antiviral activity. Overall, our results suggest that NSP5-mediated cleavage disrupts the function of CCDC50 by separating its substrate recognition and oligomerization domains, weakening its interaction with the autophagy machinery and viral targets, and thus impairing its antiviral activity.

**Fig 8:**
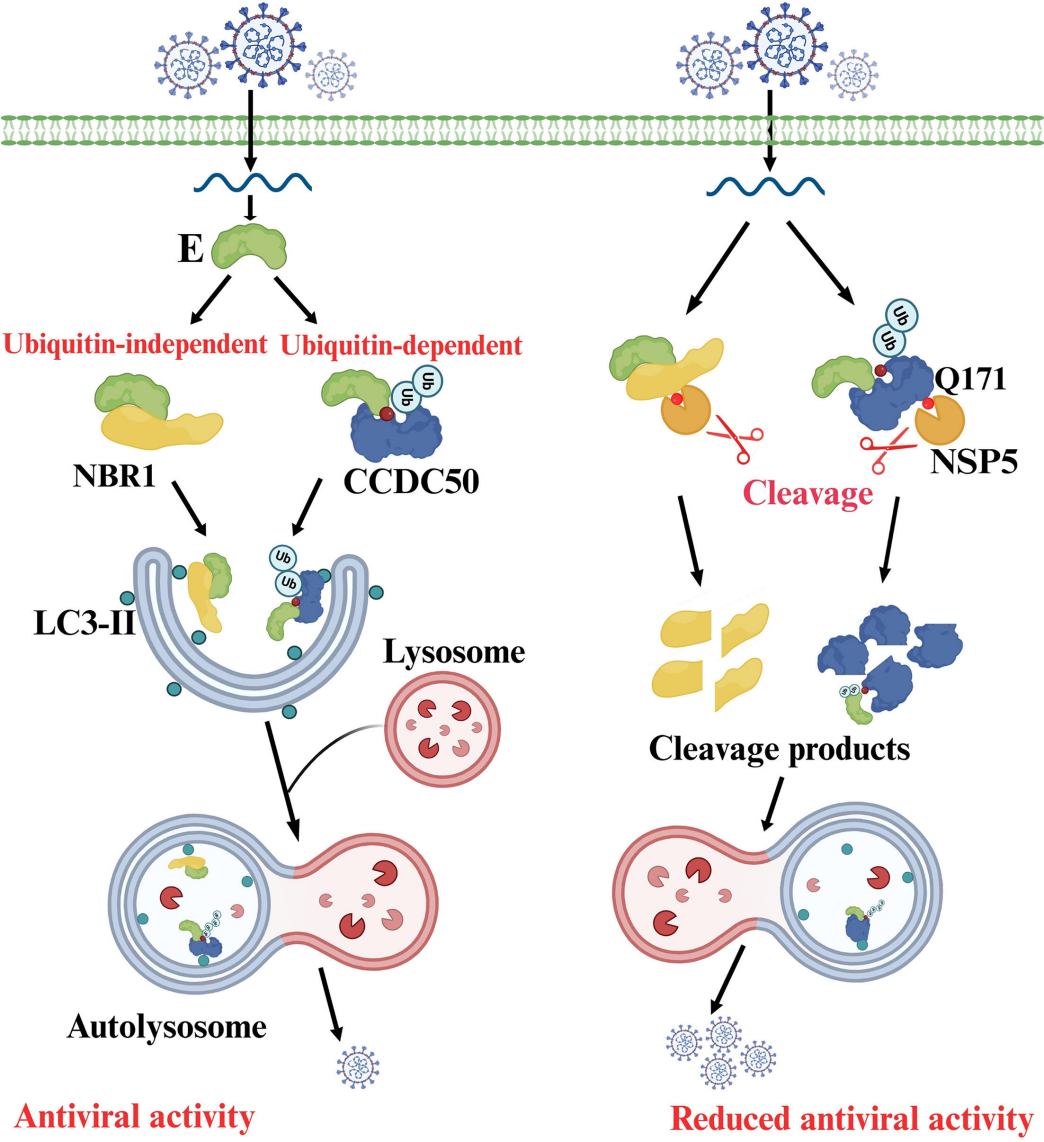
A proposed model illustrating how PDCoV NSP5 evades CCDC50-mediated antiviral responses.

In conclusion, this study identifies CCDC50 as a previously unrecognized selective autophagy receptor that restricts PDCoV replication by specifically recognizing the K63-linked polyubiquitinated E protein and promoting its autophagic degradation independently of SQSTM1 and NBR1. We also show that PDCoV evades this antiviral mechanism through NSP5-mediated cleavage of CCDC50, thereby facilitating viral replication (Fig. 8). Taken together, these results uncover a previously unknown strategy for coronavirus evasion of the immune system to subvert selective autophagy and provide mechanistic insights into host-virus interactions with potential therapeutic implications.

## MATERIALS AND METHODS

### Cells and viruses

HEK-293T (ATCC CRL-3216), HeLa (ATCC CCL-2), LLC-PK1 (ATCC CL-101), and *NBR1*-knockout LLC-PK1 cells (*NBR1*-KO; previously generated in our laboratory [19]) were maintained in Dulbecco’s modified Eagle medium (DMEM; Gibco, 12800082) supplemented with 10% fetal bovine serum (FBS; Gibco, 16000044) at 37°C and 5% CO₂. IPI-2I cells were cultured in RPMI 1640 medium (Gibco, 11875093) supplemented with 10% FBS as previously described (19). The PDCoV strain CHN-HG-2017 (GenBank: MF095123) and a recombinant GFP-expressing PDCoV (PDCoV-GFP) were propagated and stored in our laboratory (47).

### Antibodies and chemical reagents

The following primary antibodies were used: mouse monoclonal antibodies against Flag (M185-3L), HA (M180-3), GFP (M048-3), and MYC (M192-3), and rabbit polyclonal antibodies against Flag (PM020) and HA (PM032) (all from MBL International); mouse anti-CCDC50 (sc-398994, Santa Cruz Biotechnology); rabbit monoclonal antibodies against pro-caspase-3 (A19654) and caspase-3 (A19664) (ABclonal Biotechnology); rabbit anti-β-actin (AC038, ABclonal). A monoclonal antibody specific for the PDCoV N protein was produced in our laboratory, as described previously (48). Secondary antibodies included HRP-conjugated goat anti-rabbit (AS014) and goat anti-mouse (AS003) antibodies (ABclonal), Alexa Fluor 488-conjugated donkey anti-mouse IgG (R37114), and Alexa Fluor 594-conjugated donkey anti-rabbit IgG (R37119) (Thermo Fisher Scientific). Chemical reagents included 3-methyladenine (3-MA) (S2767, Selleck Chemicals), bafilomycin A1 (Baf A1) (S1413, Selleck Chemicals) and recombinant TNF-α (E7641, Selleck Chemicals), MG132 (M7449, Sigma-Aldrich), and Z-VAD-FMK (C1202, Beyotime Biotechnology).

### Plasmid construction

The full-length cDNA of porcine *CCDC50* (GenBank: XM_003132590.6) was amplified from LLC-PK1 cells and cloned into a modified pCAGGS-Flag vector (MiaoLingBio, P1267), in which a Flag tag was fused to the C-terminus of the insert. A dual-tagged construct with a MYC tag at the N-terminus and a Flag tag at the C-terminus (pCAGGS-MYC-Flag) was generated by modifying pCAGGS-Flag. Truncated mutants of CCDC50, including CCDC50-N and CCDC50-C, were cloned into the same modified pCAGGS-Flag backbone. Genes encoding the four PDCoV structural proteins (S, E, M, and N) were individually cloned into the pCAGGS-HA vector (MiaoLingBio, P0166). Site-directed mutants of the E protein (E^K39R^, E^K43R^, E^K61R^, E^K72R^, and E^K39,43,61,72R^) were synthesized by Tsingke Biotechnology Co., Ltd., and cloned into both HA-tagged and Flag-tagged pCAGGS vector. Mutations in CCDC50 (Q117A, Q122A, Q171A, and Q179A) were introduced similarly into the pCAGGS-Flag vector. The NSP5 genes from PDCoV (GenBank: MF095123), PEDV (GenBank: SNC44844), TGEV (GenBank: XEF57928), and SARS-CoV-2 (GenBank: QTC96829) were synthesized and cloned into a modified pCAGGS-HA vector (bearing a C-terminal HA tag). Catalytically inactive mutants of each NSP5 variant were generated and cloned in parallel using the same backbone. All constructs were confirmed by Sanger sequencing and transiently transfected into LLC-PK1, IPI-2I, HEK-293T, or HeLa cells using jetPRIME (Polyplus-transfection SA, 101000046). Primer sequences used for cloning are provided in Table S1.

### Generation of CRISPR-Cas9-mediated knockout cell lines

CRISPR-Cas9-mediated gene editing was used to generate *CCDC50*-KO, *SQSTM1*-KO, and *CCDC50/NBR1*-KO LLC-PK1 cells, as previously described (49). For *CCDC50*, two sgRNAs (5′-CTACGTATTATACAGCGTTA-3′ and 5′-GAATGTTCAGCGGAACCGTT-3′) were designed; sgRNAs targeting *SQSTM1* included 5′-TTCCCCGTGCTACGGCCCGG-3′ and 5′-GCGGGAGTTTGATCGCTCGT-3′. To generate *CCDC50/NBR1* double-knockout LLC-PK1 cells, sgRNAs targeting *CCDC50* (5′-GAATGTTCAGCGGAACCGTT-3′) and *NBR1* (5′-AAATACAACTTGGGCTGATG-3′) were designed. All sgRNAs were cloned into a modified pDG459 vector (Addgene #100901; gift from Paul Thomas). Cells were transfected with 2.5 μg of sgRNA constructs using jetPRIME (Polyplus-transfection SA, 101000046) and selected with puromycin (3 μg/mL) after 24 h. Single-cell clones were obtained by limiting dilution and validated by Sanger sequencing and Western blotting.

### Immunofluorescence assay (IFA)

Cells transfected with the indicated plasmids were fixed with 4% paraformaldehyde for 10 min at room temperature, washed three times with PBS (HyClone, SH30256), and permeabilized with 0.1% Triton X-100 (Sigma, T8787) for 10 min. After blocking with 5% BSA (BSA; BioFroxx, 4240GR100) for 1 h, cells were incubated with primary antibodies for 2 h at room temperature, followed by Alexa Fluor-conjugated secondary antibodies for 1 h. Cell nuclei were stained with 4′,6-diamidino-2-phenylindole (DAPI; Solarbio, C0065) for 10 min in the dark. Fluorescence images were acquired using a Zeiss LSM 880 confocal microscope.

### Flow cytometry assay

PDCoV-GFP-infected cells were harvested by trypsinization, fixed with 4% paraformaldehyde for 20 min at room temperature, and permeabilized with 0.1% Triton X-100 for 10 min. GFP-positive cells were quantified using a Beckman Coulter CytoFLEX LX flow cytometer, and data were analyzed using FlowJo v10.

### Western blotting

Cells were lysed in buffer containing 65 mM Tris-HCl (pH 6.8), 4% SDS, 3% DTT, 40% glycerol, and a protease inhibitor cocktail (Sigma, P8340). The lysates were boiled, separated by 12% SDS-PAGE, and transferred to PVDF membranes (Millipore, IPVH00010). The membranes were blocked with 5% nonfat milk in TBST (0.1% Tween-20 in TBS) for 2 h at room temperature and incubated with primary antibodies overnight at 4°C. After washing, the membranes were incubated with HRP-conjugated secondary antibodies for 1 h at room temperature. Protein bands were visualized with enhanced chemiluminescence (ECL; Bio-Rad, 1705061) and imaged using the Tanon 5200 Multi Chemiluminescent Imaging System (Tanon Science & Technology).

### Co-immunoprecipitation (co-IP)

Cells were transfected with the indicated plasmids and lysed in 500 μL of ice-cold RIPA buffer (Beyotime, P0013B) containing protease inhibitors for 30 min. Lysates were clarified by centrifugation at 13,000 × *g* for 10 min at 4°C. A portion of the supernatant was reserved as whole-cell lysate input. The remaining supernatant was incubated with the indicated primary antibodies for 8 h at 4°C with gentle rotation, followed by incubation with Protein A/G Plus-Agarose beads (Santa Cruz Biotechnology, sc-2003) for a further 4 h at 4°C. The beads were washed four times with cold lysis buffer and boiled in SDS loading buffer for 10 min. The immunoprecipitated proteins were analyzed by Western blotting.

### TCID_50_ assay

Virus-infected cells were subjected to three freeze-thaw cycles and clarified by centrifugation at 10,000 × *g* for 10 min. Viral titers were determined by the TCID_50_ method using the Reed and Muench calculation, as previously described (47). Briefly, LLC-PK1 cells were seeded into 96-well plates and inoculated with 100 μL of 10-fold serial dilutions of virus samples. After 3 days of incubation, the cytopathic effects were observed and scored to calculate TCID_50_ values.

### Quantitative reverse transcription PCR (RT-qPCR)

Total RNA was extracted with TRIzol reagent (Vazyme, R401), and reverse-transcribed into cDNA with RT SuperMix for qPCR (Vazyme, R122). RT-qPCR was performed with SYBR Green Master Mix (Vazyme, Q111). GAPDH was used as an internal reference gene, and relative mRNA expression was calculated using the 2^−ΔΔCT^ method. Primer sequences are shown in Table S1.

### Cell viability assay

Cell viability was determined using the CCK-8 assay (Beyotime, C0043). *CCDC50*-KO, *SQSTM1*-KO, *CCDC50/NBR1*-KO, and WT LLC-PK1 cells were seeded in 96-well plates. After incubation, 10 μL of CCK-8 reagent was added to each well and incubated at 37°C for 1 h. The absorbance was measured at 450 nm using a microplate reader.

### Statistical analysis

Statistical analyses were performed using GraphPad Prism (GraphPad Software, Inc.). Data are presented as mean ± standard deviations (SDs). Comparisons between groups were performed using an unpaired two-tailed Student’s *t*-test. *, *P* < 0.05; **, *P* < 0.01; and ***, *P* < 0.001.

## Data Availability

All data are available in the main text and supplemental material.

## SUPPLEMENTAL MATERIAL

Supplemental figures mbio.00259-26-s0001.pdf Fig. S1 and S2.

Table S1 mbio.00259-26-s0002.pdf Primers used for gene cloning and RT-qPCR in this study.

ASM does not own the copyrights to Supplemental Material that may be linked to, or accessed through, an article. The authors have granted ASM a non-exclusive, world-wide license to publish the Supplemental Material files. Please contact the corresponding author directly for reuse.
