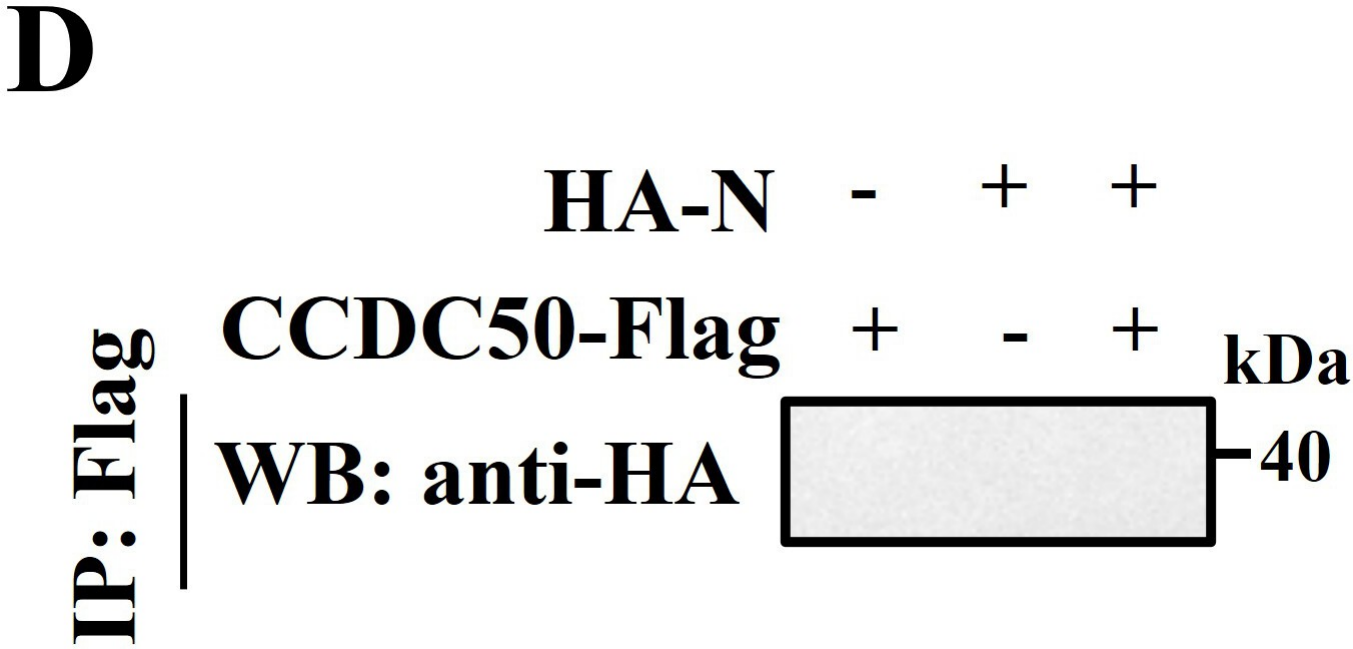# Correction for Li et al., “PDCoV NSP5 cleaves the selective autophagy receptor CCDC50 to disrupt autophagic degradation of the viral envelope protein”

**DOI:** 10.1128/mbio.01535-26

**Published:** 2026-07-17

**Authors:** Ke Li, Hongyun Wei, Kangli Zhao, Dong Chen, Yu Sun, Peng Zhou, Hui Jin, Anan Jongkaewwattana, Sizhu Suolang, Dang Wang, Hongbo Zhou, Rui Luo

## AUTHOR CORRECTION

Volume 17, no. 4, e00259-26, 2026, https://doi.org/10.1128/mbio.00259-26. Figure 4D: The anti-HA immunoblot panel corresponding to the IP samples should appear as shown in this correction. This panel was inadvertently replaced with an incorrect anti-HA immunoblot panel during figure assembly. We apologize for this error. This correction does not affect the results, interpretation, or conclusions of the article.

**Fig 4:**