# Supplementary material for: PDCoV NSP5 cleaves the selective autophagy receptor CCDC50 to disrupt autophagic degradation of the viral envelope protein
Source: mBio. 2026 Mar 12;17(4):e00259-26. doi: 10.1128/mbio.00259-26 (PMC13059773; doi:10.1128/mbio.00259-26)
Supplement: Supplemental figures — Fig. S1 and S2. [file mbio.00259-26-s0001.pdf]

Supporting information

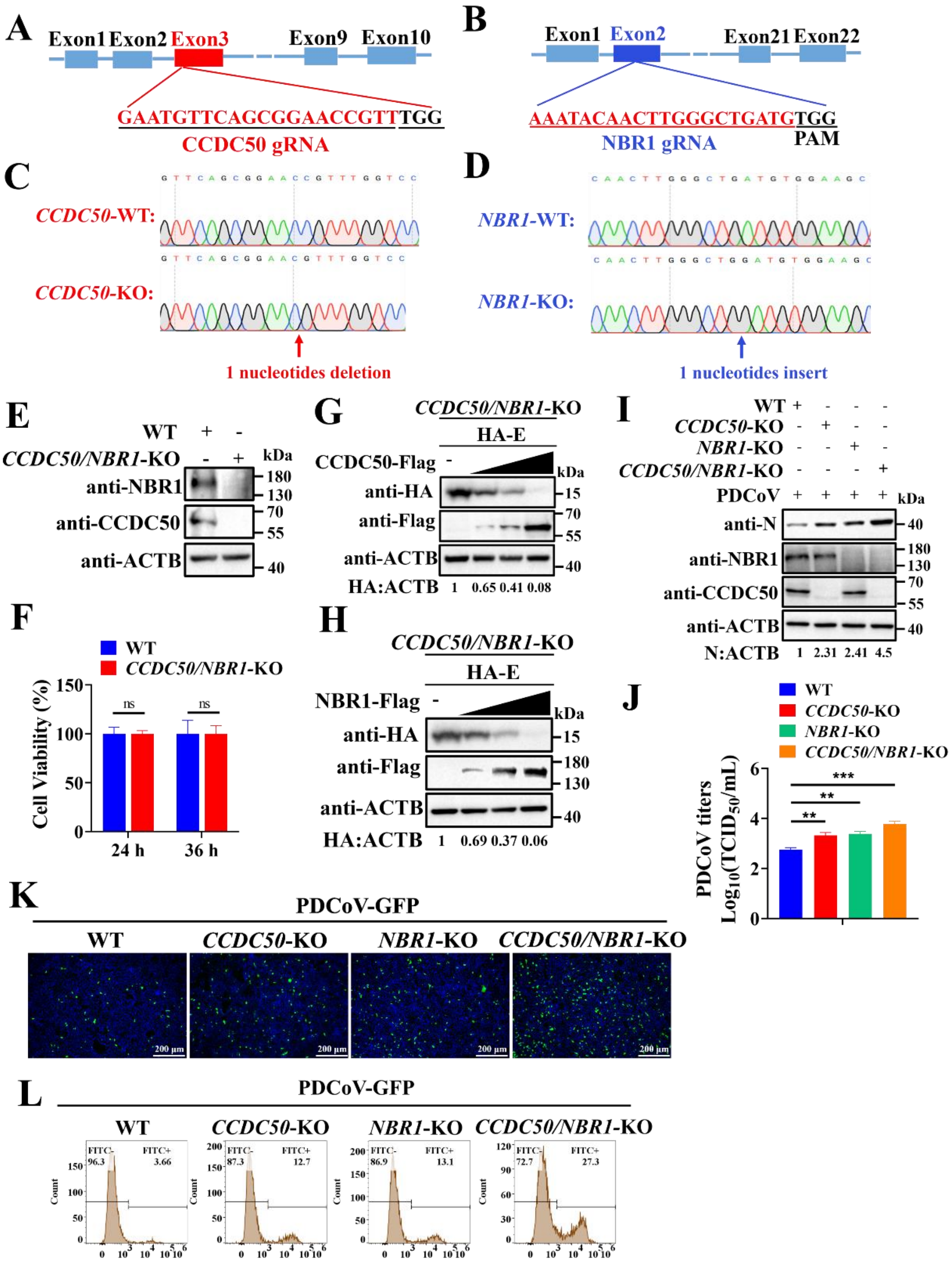

Figure S1. CCDC50 mediates autophagic degradation of the PDCoV E protein and restricts PDCoV infection independently of NBR1. (A, B) Schematic representation

of the *CCDC50*/*NBR1*-KO strategy. **(C-E)** Validation of *CCDC50/NBR1*-KO in LLC-PK1 cells by Sanger sequencing (C, D) and Western blotting (E). **(F)** Cell viability of WT and *CCDC50*/*NBR1*-KO LLC-PK1 cells. **(G, H)** *CCDC50*/*NBR1*-KO cells were transfected with increasing amounts of CCDC50-Flag (G) or NBR1-Flag (H) together with HA-E. Protein levels were determined by Western blotting. **(I, J)** WT, *CCDC50*-KO, *NBR1*-KO and *CCDC50/NBR1*-KO cells were infected with PDCoV, and samples were collected at the indicated time points for Western blotting (I) and $TCID_{50}$ assays (J). **(K, L)** WT, *CCDC50*-KO, *NBR1*-KO and *CCDC50/NBR1*-KO cells were infected with PDCoV-GFP for 7 h and analyzed by confocal microscopy (K) and flow cytometry (L). Scale bar, 200 μm. Results from cell viability (F) and $TCID_{50}$ (J) assays are presented as the mean ± SD of three independent experiments. Statistical significance was determined using Student's *t*-test. *, $P < 0.05$; **, $P < 0.01$; ***, $P < 0.001$; ns, not significant.

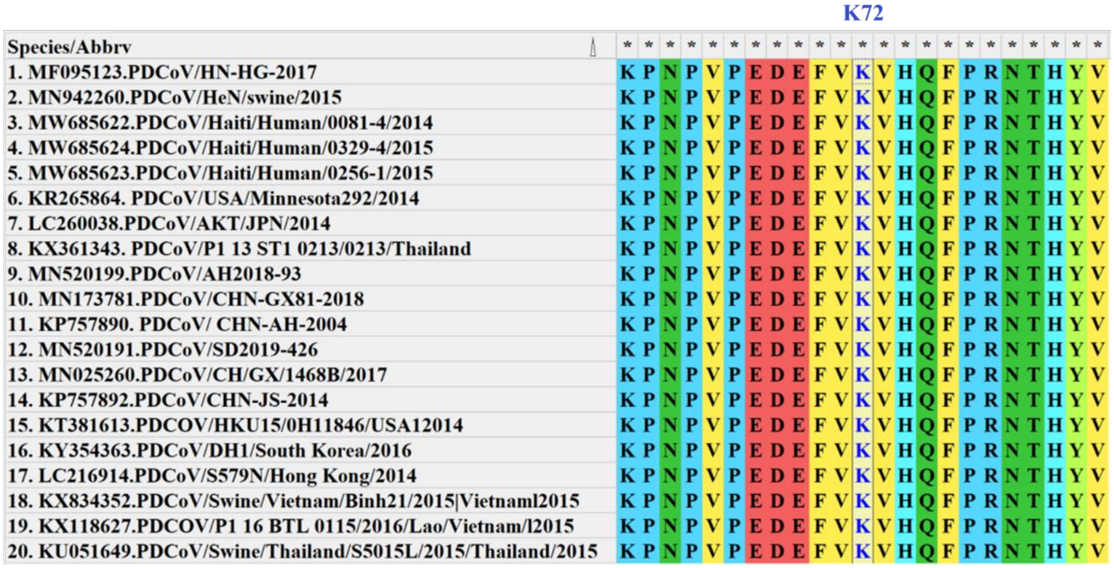

Figure S2. Alignment of porcine PDCoV E protein sequences from representative strains. Amino acid sequences from diverse PDCoV isolates were obtained from GenBank and aligned. The lysine at position 72 (K72) is highly conserved across strains.
