# Supplementary material for: PDCoV NSP5 cleaves the selective autophagy receptor CCDC50 to disrupt autophagic degradation of the viral envelope protein
Source: mBio. 2026 Mar 12;17(4):e00259-26. doi: 10.1128/mbio.00259-26 (PMC13059773; doi:10.1128/mbio.00259-26)
Supplement: Table S1 — Primers used for gene cloning and RT-qPCR in this study. [file mbio.00259-26-s0002.pdf]

Table S1. Primers used for gene cloning and RT-qPCR in this study.

Name	Forward sequence (5′-3′)	Reverse sequence (5′-3′)
CCDC50	ATGTCTGAAGTCAGCATCGACC	TGGCTTATAATGGAAACCTTTATGAG
CCDC50^{Q117A}	CATAGCTCGACTCTTGGCAGAAAAGGAGTTAC	GTAACTCCTTTTCTGCCAAGAGTCGAGCTATG
CCDC50^{Q122A}	GCAAGAAAAGGAGTTAGCGGAAGAGAAAAAGC	GCTTTTTCTCTTCCGCTAACTCCTTTTCTTGC
CCDC50^{Q171A}	GTCTTGTAGACTCGCAAGCGAGGAGAAGAC	GTCTTCTCCTCGCTTGCGAGTCTACAAGAC
CCDC50^{Q179A}	GGAGAAGACTGTGAAGGCGAGGAAGGAGAAATG	CATTTCTCCTTCCTCGCCTTCACAGTCTTCTCC
CCDC50-N	ATGTCTGAAGTCAGCATCGACC	TTGGAGTCTACAAGACCTTGAG
CCDC50-C	AGCGAGGAGAAGACTGTGAAGC	TGGCTTATAATGGAAACCTTTATGAG
PDCoV-E	CTGAATTCATGGTAGTCGACGAC	TAGCTCGAGTCAGACATAGTGAGTG
PDCoV-M	CTGAATTCGAGCTCATGTCTGAC	TAGCTCGAGTTACATATACTTATACAGGCG
PDCoV-N	CTGAATTCATGGCTGCACCAGTGG	AGCTCGAGCTACGCTGCTGATTCC
PDCoV-S	CTGAATTCATGCAGAGAGCTCTATTG	CTGCTAGCCTACCATTCCTTAAAT
qPCR-CCDC50	TCAAGGAAGTGTGCCGAGAT	CGATGCCAAGTGATGCTCAA
pig GAPDH	ATGGTGAAGGTCGGAGTGAAC	GGCGACAATGTCCACTTTGC
SQSTM1	ATGAGGGGCGGGGCTTCACC	TCACAAGGGCGGTGGGTGTTT
